# Guided by Energy, Ethics and Empathy

**DOI:** 10.1177/14550725251396063

**Published:** 2025-11-20

**Authors:** Matilda Hellman

**Affiliations:** 1 Department of Sociology, 225313 Uppsala University, Uppsala, Sweden

**Keywords:** drugs, drug-related death, publishing, recovery, comorbidity

In late August, the editorial team received devastating and saddening news. Our journal’s *grand old lady*, Kerstin Stenius, had passed away. It is impossible to overstate the role Kerstin has played for this journal and for our field of research. We wanted to dedicate this issue to her memory, but that feels redundant, since everything we do at the editorial office is, in one way or another, already an homage to her.

Kerstin has shaped our understanding of what NAD is as a scientific journal and as a product. She has formed our sense of quality and our sense of boundaries regarding the journal's mission: how we work with texts, how we read submitted manuscripts and how we relate to the research field that produces the material we publish. Kerstin was someone who could see connections and overarching patterns in ways that created continuity. Yet, she was never afraid to think differently or propose bold new directions.

Her passing confirmed what we in fact already knew: she had built a wide circle of “fans” across the world. Through her research projects, at conferences and seminars, and through her involvement in organisations such as KBS (Kettil Bruun Society for Social and Epidemiological Research on Alcohol), ISAJE (International Society of Addiction Journal Editors) and ICARA (International Confederation of Alcohol, Tobacco and other Drug Research Associations), she had over the years gathered a remarkable number of colleagues and friends who appreciated her in an unusually warm and engaged way. We have collected tributes from some of these colleagues in an In Memoriam section of this issue of NAD (Hellman et al., 2025).

This issue, rich and multifaceted, is a suitable frame for our tribute. We conclude our publication year 2025 with, among other things, a comprehensive comparative study on risks and resources among people who use drugs in Denmark and Sweden (Houborg et al., 2025). Fagerström et al. (2025) explore how support for concerned significant others is shaped by professionals’ understandings of alcohol and other drug problems in Sweden. We also present a study on how recovery pathways in Sweden are associated with time, gender and meaningful activities (Nisic et al., 2025). Comorbidity and co-occurring problems are addressed in an article on gambling by a Finnish research team (Salonen et al., 2025) and by a Swedish team in relation to substance use (Kapetanovic et al., 2025).

In a separate thematic section with its own framing (Karjalainen et al., 2025), guest editors Karoliina Karjalainen, Sanna Rönkä and Pekka Hakkarainen have gathered Nordic studies on drug-related mortality. Scarpa et al. (2025) report findings from Swedish Addiction Severity Index (ASI) data with a focus on educational attainment and deaths of despair among individuals assessed for substance use severity. O’Gorman et al. (2025) present a socio-ecological autopsy study from a Scottish region. Pitkänen et al. (2025) analyse age-specific mortality and causes of death among men, women and accompanying children following substance use treatment. The dual experience of loss and drug use is discussed in an article by Selseng et al. (2025).

Given the breadth of contributions, it has also been fitting to add commentaries (Bilgrei & Hanoa, 2025; Savonen, 2025) that we hope will carry the discussions forward from the pages of the journal into research environments and the wider society.

We enter the year 2026 with the hope of continuing to publish work that meets the vision and the standards of excellence that Kerstin Stenius developed and upheld for so many years.

This is something that one of Kerstin's oldest friends, Pia Rosenqvist describes with three Es – energy, ethics and empathy (Hellman et al., 2025) We shall let these words lead our work!
